# Psychometric Validation of the Portuguese Obsession with COVID-19 Scale (PT-OCS)

**DOI:** 10.3390/healthcare12050563

**Published:** 2024-02-28

**Authors:** Mónica Taveira Pires, Susana Mourão, José Santos

**Affiliations:** 1 Psychology Research Centre (CIP-UAL), Universidade Autónoma de Lisboa, 1169-023 Lisbon, Portugal; smourao@autonoma.pt; 2 Beatriz Ângelo Hospital, 2674-514 Loures, Portugal; jose.santos3@hba.min-saude.pt; 3 Medicine Faculty, Universidad de Extremadura, 06006 Badajoz, Spain

**Keywords:** COVID-19 obsession scale, confirmatory factor analysis, Portuguese validation, general sample, parents

## Abstract

The psychological effects of the COVID-19 pandemic on the Portuguese population are quite relevant and partially related to their repetitive and disruptive thinking about the disease. The successive periods of lockdown contributed to an additional burden on the family–work–life balance for parents. This study aims to validate the Portuguese version of the Obsession with COVID-19 Scale (PT-OCS), which was developed using a general sample from several regions of the country (*n* = 531) together with a specific sample of Portuguese parents (*n* = 109). The confirmatory factor analysis results indicate that the PT-OCS includes a set of excellent psychometric properties concerning both the general sample (*χ*^2^_(1)_ = 0.446, *p* = 0.504; CFI = 1.0; GFI = 1.0; RMSEA = 0.0; standardised RMR = 0.003) and the parent group (*χ*^2^_(2)_ = 1.816, *p* = 0.403; CFI = 1.0; GFI = 0.99; RMSEA = 0.0; standardised RMR = 0.016; Bollen–Stine bootstrap *p* = 0.65). The scale shows very good reliability (0.84 < α/ω < 0.88). As expected, obsession with COVID-19 was highly correlated with COVID-19 anxiety, and women had higher PT-OCS scores. The findings suggest that the PT-OCS is a reliable and valid measure for both persistent and disruptive thinking about COVID-19 in different groups of the Portuguese population, with potential for studying future epidemic events.

## 1. Introduction

Coronavirus disease 2019 (COVID-19) was first discovered in China in December 2019 and spread worldwide quickly, reaching global pandemic status by March 2020 [1]. By May 2023, the number of deaths due to COVID-19 reached 6,927,378, with a total of 765,903,278 infected cases documented [2]. In Portugal, COVID-19 has killed 26,616 registered people and infected 5,582,987 more [2].

As COVID-19 is highly transmissible among humans, several restrictive measures were adopted, including quarantine in cases of suspected infection and physical isolation for infected or symptomatic people [3]. In Portugal, two states of emergency and lockdown periods were declared (March–May 2020; January–April 2021), with implications for freedom of movement, physical distancing, and social contact. Several economic, religious, sporting, and cultural activities had to be shut down, and school and work activities transitioned to remote teaching and remote working whenever and wherever possible. 

The disease itself had deep effects on everyday life, amplified by the lockdown measures imposed to control the spread of the virus. Consequently, relevant psychological effects impacted the general population. Several review papers have addressed the negative psychological symptoms resulting from the pandemic, including post-traumatic stress, intense feelings of frustration and anger, sleep disturbance, uncontrolled fear, and obsessive behaviours [4,5,6,7]. We also found low levels of psychological well-being and high levels of depression and anxiety [8,9,10], compared to results from previous research on COVID-19 [11]. Some of these problems have been highlighted to be connected to exposure to overwhelming, contradictory, and sometimes misunderstood information about COVID-19, which was continually widely spread on social media [3,5,12], fuelling repeated and disruptive thoughts concerning the pandemic. Individuals with a history of mental health issues, such as anxiety, obsession, adjustment disorders, or obsessive traits, were found to be more likely to have severe lingering consequences after the height of the pandemic [13,14].

Psychological impacts from the COVID-19 outbreak have been found specifically in the Portuguese general population, namely moderate to severe stress [15,16]. Other evidence from previous research indicates a normal range regarding the status of mental health in the Portuguese population [17], identifying sociocultural differences in the overall impact of the pandemic on mental health [18,19,20]. Indeed, both internationally as well as in Portugal, some particular groups have been consistently identified to be at a greater risk for psychosocial vulnerability; namely, women (due to an overload of work and household chores), people with lower socio-economic status, discriminated communities, health professionals and other frontline workers, people with previous (mental) health conditions, and those with barriers to accessing healthcare [5,8,11,16,21].

Although less-studied, parents or caregivers of children were also more prone to developing psychological problems during the COVID-19 pandemic [15], based on the fact that many families experienced multiple hardships during this crisis (e.g., job or income loss, home-schooling and disrupted routines, less social support, and caregiving burden) [22,23,24,25]. Consequently, some evidence points to increased levels of negative moods, higher parental conflict, psychological distress, and anxiety states, mainly associated with difficulties related to multiple obligations and conflicting responsibilities [22,26,27]. Ultimately, parents’ perceptions of COVID-19 may have harmed their children’s behaviours and wellbeing [28,29]. 

In the aftermath of the pandemic, after mass vaccination, social contact is still being restored, and there is less available information about COVID-19 as the disease is officially being managed as an endemic entity. Nevertheless, it is of utmost importance to continually assess people’s concerns about COVID-19 (especially vulnerable groups), as some of the psychological effects may linger over time, as has happened during other—although smaller-scale—epidemic events (e.g., the Ebola and 2003 SARS-CoV outbreaks) [3,4,7]. It is important to highlight that even prior to COVID-19, nearly 17.3% of Europeans were already managing mental health issues, especially anxiety disorders, and Portugal presented a higher psychological distress rate (20% vs. 11% in EU). As a country, Portugal has presented the worst mental health burden indicators, as well as the highest-ranking percentage of people in mental distress [30]. In April 2020, during the first wave of COVID-19, a phone line for psychological support was issued by the National Health Service (NHS, SNS in Portugal) in collaboration with the Portuguese Psychologist Association/Order (OPP). This was an important measure for psychological health. More serious identified cases were referred to ER services, and cases of psychological suffering were sometimes directed to the SNS24 phone line. This line was used to work on referrals to the NHS. Psychological teams from both the NHS and non-government organisations also provided either individual or group psychological support for healthcare workers. Despite this, a shortage of psychologists is still observed in the NHS, with implications for access to assessment and, consequently, to the earlier detection of maladjustments, such as depression, anxiety, and obsession, as well as psychological support. 

Among other psychological distress symptoms, obsession with COVID-19 has emerged as a specific anguish and mental health maladjustment. Persistent dysfunctional COVID-19 thinking patterns, dreams, and fear of contagion, tied to clinical anxiety and functional impairment in everyday life or maladaptive coping strategies (e.g., alcohol or drug consumption), are the main aspects of this obsession [31]. High levels of obsession with COVID-19 are also strongly associated with coronavirus anxiety, spiritual crisis, hopelessness, and suicidal ideation, suggesting the existence of more severe mental disorders that need to be addressed [31]. Repeated exposure to the virus (e.g., for frontline workers), COVID-19 information overload, and social isolation were possible triggers for obsessive thoughts, which may have resulted in psychological distress, maladjustment, or mental disorders [32].

The Obsession with COVID-19 Scale (OCS) [31] was developed at the beginning of the pandemic and later adapted and validated in several countries worldwide (Brazil [32]; Peru [33]; China [34]; Republic of Korea [35]; Japan [36]; Turkey [37]; Iran [38]; and specific ethnic groups [39] and professional groups, such as frontline health staff [40]), with proven psychometric properties for clinical screening. Accordingly, this study aims to validate the Portuguese version of the Obsession with COVID-19 Scale using a general sample from all regions of the country, as well as a more specific sample of Portuguese parents.

## 2. Materials and Methods

### 2.1. OCS Translation and Cultural Adaptation

Following some of the recommendations of Beaton et al. [41] regarding the cross-cultural adaptation of health status measures, three independent bilingual translators translated the OCS from English to European Portuguese. Afterwards, it was back-translated by another bilingual translator, who also resolved discrepancies in the translated and back-translated versions. This resulted in the final version of the PT-OCS [42]. 

#### OCS Validation Plan and Data Analysis

The authors of this cross-sectional study analysed the psychometric properties of the PT-OCS based on international guidelines for psychometric testing [43,44]. More specifically, the underlying factor structure of the PT-OCS was confirmed using confirmatory factor analysis (CFA) with maximum likelihood (ML) estimation, carried out using AMOS 28.0 [45]. To ensure that the proposed unidimensional structure presented an adequate fit for the sample data, the following goodness-of-fit indices were used: normed chi-square (*χ*^2^/*df*), with an acceptable fit of <5; the comparative fit index (CFI) and goodness-of-fit index (GFI), estimating a good fit of >0.9; a root-mean-square error of approximation (RMSEA) of ≤0.06 and standardised root-mean-square residual (SRMR) of <0.08 for good fit [46,47,48]. Evidence of its reliability was assessed using the Cronbach’s alpha and MacDonald’s omega coefficients [49], for which values higher than 0.7 indicate satisfactory internal consistency and reliability [44,50]. Additional evidence of the validity of the OCS was determined through calculating the associations between the PT-OCS and the Coronavirus Anxiety Scale (CAS) [51] scores. Based on previous OCS validations, we expected middle-to-high correlations between these two indicators of psychological stress during the pandemic (H1). Criterion-related validity was confirmed according to differences in the PT-OCS scores between males and females, with women expected to have higher levels of anxiety and obsession (H2).

### 2.2. Measures

#### 2.2.1. Demographic Sheet

We used a form to request the participants’ background information (e.g., sex, age, educational level, marital status). In the general population, specific indicators of the participants’ socio-economic and living conditions status were assessed based on the Graffar Index (profession, family income, housing, and neighbourhood conditions), as they may be relevant mental health risk factors, especially during the COVID-19 confinements [4,5,16]. This index describes people’s sociodemographic conditions in different settings and populations, helping to elucidate the living conditions and contextual variables relevant to health indicators. In the case of the parent group, we also asked for socio-demographic information about their children (sex, age, school attendance, number of brothers/sisters). The data were coded to ensure confidentiality and anonymity. 

#### 2.2.2. Coronavirus Anxiety Scale (CAS)

The CAS is a brief mental health measure developed in the context of the pandemic to identify dysfunctional anxiety symptoms specifically associated with coronavirus (dizziness, sleep disturbance, tonic immobility, appetite loss, abdominal distress [51]). The 5 items are rated on a 5-point Likert scale regarding the perceived frequency of the symptoms in the preceding two weeks, ranging from 0 (not at all) to 4 (nearly every day). Scores equal to or higher than a cut-off point of 9 indicate dysfunctional levels of COVID-19 anxiety.

#### 2.2.3. Obsession with COVID-19 Scale (OCS)

The OCS assesses four indicators, presented as items, of persistent and dysfunctional thinking regarding COVID-19, which may be associated with anguish and mental distress (having disturbing thoughts that oneself or someone else has caught the coronavirus, dreaming, and repetitively thinking about the coronavirus [31]). Each item is rated on a 5-point scale, reflecting the perceived frequency of the above-mentioned thoughts in the preceding two weeks from 0 (not at all) to 4 (nearly every day)). Total OCS scores equal to or higher than 7 suggest the presence of intrusive, repeated obsessional thoughts about COVID-19.

### 2.3. Procedure and Ethical Clearance

Participants were recruited using a snowball approach, with support from community institutions, hospitals, and virtual platforms. This study was approved by the Psychology Research Centre ethics committee (approval number 8/2020). All participants filled out a paper or online survey after being informed about the study’s aims, voluntary participation, confidentiality, and anonymous status of the data, giving their consent to participate. Each participant was also given an email address to which they could send and clarify any possible questions or requests for the research outcomes. All the information was anonymous, coded, and securely stored for the required period. The statistical reports did not involve any identifying data.

## 3. Results

### 3.1. Sample Characteristics

The general sample was composed of 531 participants (78% women), 68.2% of whom were in the age group of 21 to 49 years old. Nearly half were in a marital relationship (39% married and 13.6% living together as a couple). The majority reported professional (79.1%) or school involvement (12.4%), and 11.3% were self-employed. Our sample covered all regions of the Portuguese territory, namely the 18 administrative districts and the 2 autonomous regions/archipelagos (Madeira and Azores). Beja, a rural south area, and Lisbon, the country capital, were the most represented regions (37.5% and 36.2%, respectively). Most of the participants reported living in an urban context (71.8%). The general sample had heterogeneous socio-economic conditions; although, it mostly represented a middle-to-high socioeconomic status. Approximately half of them had university degrees (49.3%), and 26.7% had nine years of schooling. A total of 79% of the participants earned a monthly income, and only 16.6% had task-based incomes. Most participants (54.2%) indicated spacious and comfortable living arrangements, mostly in good residential quarters (76%); although, 38.6% reported more modest household conditions, with 15% living downtown in narrow and old streets.

The second sample was part of a larger pool of data collected as part of a transcultural research project running during the pandemic and aiming to explore the effects of different (co)parenting determinants on child psychosocial adjustment. This parent group was composed of 109 participants (73.4% mothers), aged between 24 and 59 years old (*M* = 41.5, *SD* = 7.9), of whom 31.2% were in a marital relationship. A total of 65.2% had university degrees, and 28.4% had 12 years of schooling. They had, on average, only one child, with heterogeneous ages ranging from 2 to 18 years old (*M* = 10.5, *SD* = 4.8). All the children were attending educational facilities or schools, including kindergartens (16.5%).

### 3.2. Item Sensitivity and Distributional Properties

The descriptive statistics of each of the PT-OCS items are presented in Table 1 with the summarised items. All items in both samples include the full range of possible answer values, with the lowest mean values for item 4 (I dreamed about the coronavirus) and the highest for item 2 (I had disturbing thoughts that certain people I saw may have the coronavirus).

In the general sample, no OCS item showed absolute values of skewness or kurtosis indicative of strong deviations from the normal distribution. In the parent group, item 4 is distributed slightly skewed (*ku* > 7 [50]). Accordingly, in the confirmatory factor analysis results that follow, a bootstrap resampling method (200 samples, 95% CI) was used to validate the solution obtained using the ML method.

### 3.3. OCS Dimensionality and Reliability

**Figure 1:**
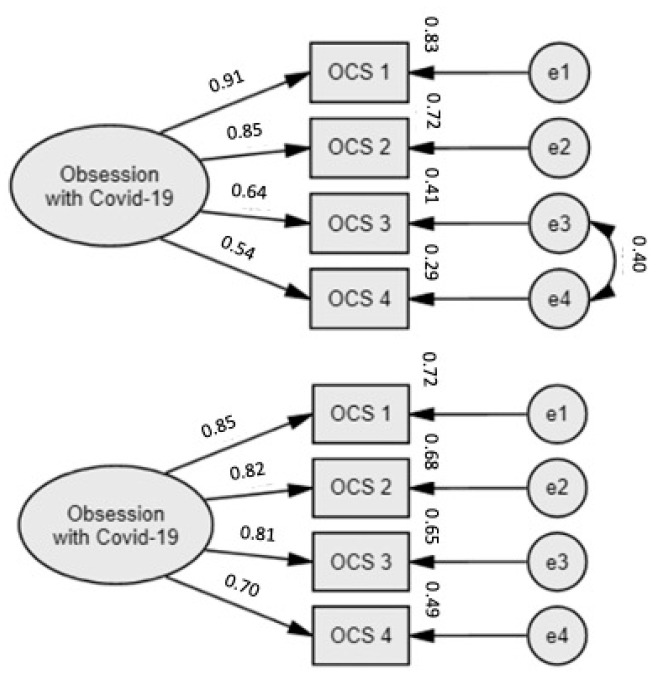
Single-factor structure of the PT-OCS with four items, loadings, and errors for both the Portuguese general sample (*n* = 531) and the Portuguese parent group (*n* = 109). Standardised loadings are shown. All items loaded significantly on the corresponding factor.

The results of the confirmatory factorial analysis support that the items of the PT-OCS are coherent together in a single-factor model (Figure 1).

The presented model shows a good fit, with excellent fit indices, for both the general sample (*χ*^2^_(1)_ = 0.446, *p* = 0.504; CFI = 1.0; GFI = 1.0; RMSEA = 0.0; standardised RMR = 0.003) and the parent group (*χ*^2^_(2)_ = 1.816, *p* = 0.403; CFI = 1.0; GFI = 0.99; RMSEA = 0.0; standardised RMR = 0.016; Bollen–Stine bootstrap *p* = 0.65, corrected with a resampling bootstrap). 

The reliability coefficients for the entire four-item scale are considered very good indicators of the measure’s internal consistency, for both the general sample (*α/ω* = 0.84) and the parent group (*α* = 0.87; *ω* = 0.88). 

### 3.4. Correlations and Criteria-Related Validity

As expected, and supporting the additional validity of the PT-OCS (H1), high positive correlations were found between the OCS and the CAS scores, for both the general sample (*r* = 0.76, *p* < 0.01) and the parent group (*r* = 0.80, *p* < 0.01). As hypothesised (H2), the women’s PT-OCS mean scores indicate higher levels of obsession with COVID-19 (Table 2) in both the general sample (*t*_(220.02)_ = − 4.02, *p* < 0.001) and the parent group (*t*_(85)_ = 2.28, *p* = 0.025). 

In the general sample, the PT-OCS scores also differed based on the participants’ ages (*r* = −0.16, *p* < 0.01), as well as on some indicators of socio-economic and living condition status: (i) school level (*rs* = 0.15, *p* < 0.01); (ii) profession (*F*_(3, 527)_ = 204.605, *p* = 0.002; higher mean scores for less specialised workers); and (iii) house conditions (*F*_(2, 528)_ = 176.314, *p* = 0.002; higher mean scores for less suitable accommodations).

### 3.5. PT-OCS Scores and Clinical Cut-Off Points

When applying the criteria for an optimal cut-off score of ≥7 for the OCS [31], we estimated that 23.2% of Portugal’s general population reported excessive levels of obsession with COVID-19 (high COVID-19 obsession group); 46.3% of them also indicated high COVID-19 anxiety (cut-off score of ≥9 for CAS [51]; *χ*^2^_(1)_ = 165.123, *p* < 0.001). Only 10.1% of the parents were included in this high COVID-19 obsession group, of which 36.4% of them also indicated dysfunctional levels of COVID-19 anxiety (Fisher’s exact test, *p* < 0.001).

Following the above trends, the COVID-19 obsession group from the general population sample (above the recommended cut-off point vs. the non-obsessive group) comprised more women than men (85.4% vs. 75.7%, *χ*^2^_(1)_ = 5.102, *p* = 0.024). When compared, this highly obsessive group also represented a greater prevalence of non-specialised professionals (24.4% vs. 15.4%; *χ*^2^_(3)_ = 9.794, *p* = 0.02), widely working as frontliners (e.g., drivers, assistants, cleaners), and people who lived in less-suitable accommodation (7.3% vs. 2.7%; *χ*^2^_(2)_ = 7.365, *p* = 0.025). Additionally, in the general population sample, a higher percentage of participants with a low level of education were part of this high-COVID-19-obsession group when compared to the non-obsessive group (*χ*^2^_(3)_ = 9.558, *p* = 0.023). Parents with high COVID-19 obsession tended to have older children (*M* Rank = 73.14 vs. 52.96, *U* = 738.500, *p* = 0.044). 

## 4. Discussion

The aim of this study was to validate the PT-OCS, which is of utmost importance. To the best of our knowledge, there are currently no tools to assess persistent and disruptive thoughts about COVID-19 in the Portuguese population. The results demonstrated that the single-domain four-item scale shows robust psychometric properties, along with solid internal consistency, good construct validity, and reliability. Accordingly, our work supports the existence of a new mental health scale to be used after the pandemic to longitudinally assess obsessive thinking about COVID-19 or other potential epidemic diseases in the Portuguese population. This measure can be helpful for clinical screening and earlier detection of severe mental health disorders. Indeed, some evidence points to increasing odds of observing other pandemics or outbreak events like COVID-19 over the coming decades, especially due to the occurrence of unprecedented environmental and demographic changes (e.g., air travel globalisation, population growth, and city densities), which usually increase the emergence of diseases from zoonotic reservoirs [52,53]. 

Overall, the Portuguese population follows the trends in other cultural contexts, including Portuguese-speaking countries (e.g., Brazil) and others (e.g., Republic of Korea, Peru), where the highest mean values were also shown for item 2 (I had disturbing thoughts that certain people I saw may have the coronavirus) and the lowest for item 4 (I dreamed about the coronavirus) [32,33,35]. Nevertheless, a much higher percentage of the general Portuguese population presented dysfunctional COVID-19 thinking, namely when compared to similar samples from China and the Republic of Korea [34,35]. The prevalence of COVID-19 obsession in the Portuguese sample was only similar to that in a specific police and military sample from Peru, whose mental health problems tended to be common, even prior to COVID-19 [33,54]. This conclusion reinforces the pertinence of monitoring the mental distress associated with the pandemic in the Portuguese population, even after its official end.

Although the answer trends were similar in both analysed groups, the parents had less COVID-19 obsession. This could be explained by circumstantial events in the data collection procedures, as the general population answered the protocol specifically during a confinement period (March–April 2021), whereas the recruitment concerning parents included confinement and non-confinement periods. Although caregivers of children were more prone to psychological burden during the pandemic [15,22,23,24,25], in this case, increasing multiple and time-consuming parental responsibilities may have ended up minimising their exposure to pandemic information and news content, thus acting as a protective factor against obsessive thinking about COVID-19. These findings point to a possible protective factor of family against stressors, as it could act as a distractor and buffer effect due to multitasking demands and social support. This hypothetical justification should be confirmed in future research.

In line with previous research, the PT-OCS scores were positively correlated with COVID-19 anxiety [31,32,35], showing that dysfunctional thinking about the disease may be strongly tied to other mental health vulnerabilities and, thus, to the poorest mental health state in general. The results also confirm interesting patterns relative to the groups at risk for mental health issues related to COVID-19 [5,8,16,21], some of which have also been found in other studies on the transcultural validation of the OCS [31,32,34,35], correlating coronavirus anxiety with the path to burnout among frontline healthcare workers [40]. For instance, COVID-19 obsession was higher among women, young people, those with lower socio-economic status, and less favourable living conditions. In our case, results of the school level groups were not consistent with all previous studies. Indeed, in some circumstances, like our own work, a low school level was identified as a risk factor for the worst mental health regarding the pandemic outbreak [16]. However, other evidence points to more psychological stress during the lockdown periods in people with a higher educational level [15], raising questions regarding the interaction mechanisms between educational level and mental distress during pandemic or epidemic events. The PT-OCS may help to clarify these mechanisms. 

Overall, the mentioned results highlight the importance of designing interventions that target individuals or groups at greater risk of developing obsessive thoughts about COVID-19, such as women, young people, and those with more precarious jobs or housing conditions. Accordingly, this study also provides useful insights for government officials, psychologists, and health professionals interested in promoting mental health in the Portuguese population after the pandemic or during other potential epidemic events. 

This information highlights the importance of discussing both disease and health as a continuum. Beyond mitigation measures, policymakers should focus on careful health prevention actions at the individual, group, and organisational levels.

In addition, government entities need to promote mental health as a core aspect of health on a national scale, particularly more preventive and psychoeducational initiatives, as well as universal access to mental health care. There is a shortage of psychologists in the NHS, with implications for access to assessment and consequences for the earlier detection of maladjustments, such as depression, anxiety, and obsession, as well as psychological support. The recommended ratio of psychologists per inhabitant, which is an objective set by the government, is one psychologist for every 5000 citizens. However, the real numbers fall far below that: in 2022, the ratio was 1 per 9687 and, if we consider only those working in the primary health care system, the ratio is 1 per 41,188 citizens. Beyond preventive measures, this means that only 20% of the population has access to psychological care, meaning that every psychologist has to monitor 1000 people. It is worth noting that, in reality, additional needs due to the aftermath of COVID-19 and the general aggravation of mental health have not been considered [55]. More psychologists are needed in the NHS and in other government services (e.g., schools) for assuring mental care access for the general population, namely for vulnerable groups with financial restraints, who have been identified as more prone to psychological distress, maladjustment, and illness, such as obsessive disorders.

Apart from its empirical and clinical contributions, this study also has some limitations. Obsessive thinking about COVID-19 was only assessed using a self-reported measure, which may be prone to a certain level of social desirability and memory recall bias. This brief four-item measure has good internal consistency and reliability for this type of obsession, making it helpful for many health contexts. Although it is useful in the clinical mental health screening process, it cannot provide other important clinical data. When assessing more complex mental health constructs, it should be added to other measures and observations. Despite our efforts to obtain a comprehensive and heterogeneous sample, including a specific group of Portuguese parents, our participants mostly represent middle-to-high socio-economic conditions and, in the case of the parent group, people with access to internet resources. In both analysed samples, there was also a higher representation of women, despite it being a sampling condition in other OCS validation studies (see, e.g., [32,34]). It would be useful for future studies to assess the PT-OCS scores of clinical samples or people with high psychosocial vulnerability (e.g., low socioeconomic status, risky professions, and previous mental health problems).

## 5. Conclusions

This study provides a Portuguese version of PT-OCS: a reliable, valid, and parsimonious psychometric instrument which is useful for measuring persistent and disruptive thinking about COVID-19 or other potential epidemic diseases in the future. This may help researchers and clinicians to identify individuals carrying dysfunctional COVID-19 thoughts, in order to refer them for clinical intervention. Additionally, it can be used to better cluster high-risk professional or social groups, who would particularly benefit from developing more efficient disease-related coping and self-regulation strategies during outbreaks. 

## Figures and Tables

**Table 1:** Descriptive statistics of PT-OCS (*N* = 640).

	General Sample (*n* = 531)						Parent Group (*n* = 109)					
	*M*	*SD*	*Min*	*Max*	*Sk*	*Ku*	*M*	*SD*	*Min*	*Max*	*Sk*	*Ku*
1. I may catch the coronavirus	1.17	1.18	0	4	0.69	−0.52	0.68	0.94	0	4	1.70	3.05
2. Others I saw may have the coronavirus	1.34	1.20	0	4	0.61	−0.54	0.75	1.02	0	4	1.37	1.17
3. Repetitive thinking about the coronavirus	1.06	1.17	0	4	1.05	0.30	0.51	0.99	0	4	1.93	2.84
4. Dreaming about the coronavirus	0.55	0.97	0	4	1.81	2.51	0.30	0.78	0	4	2.93	8.45

**Table 2:** PT-OCS scores by participants’ sex: criteria-related validity.

	PT-OCS Mean Scores (*SD*)	
Participants’ Sex	General Sample (*n* = 531)	Parent Group (*n* = 109)
Women (*n* = 494)	4.43 (3.83) ***	2.58 (3.46) *
Men (*n* = 146)	3.03 (3.19) ***	1.34 (2.02) *

Note: * *p* < 0.05, *** *p* < 0.001.

## Data Availability

The data supporting the presented findings are available from the corresponding author upon reasonable request.
